# Effects of Full Inhalation of Sevoflurane and Total Intravenous Anesthesia on Hemodynamics, Serum Myocardial Enzymes, and Myocardial Markers in Elderly Patients Undergoing Hysterectomy

**DOI:** 10.1155/2021/9983988

**Published:** 2021-06-25

**Authors:** Xing Lan, Dong Yang, Shengnan Xie, Zhenghua Zhao

**Affiliations:** ^1^Department of Anesthesiology, Union Hospital, Tongji Medical College, Huazhong University of Science and Technology, Wuhan, Hubei 430022, China; ^2^Department of Public Health, Wuhan University of Technology Hospital, Wuhan, Hubei 430022, China

## Abstract

**Objective:**

To compare the effects of sevoflurane inhalation and intravenous anesthesia on hemodynamics, serum myocardial enzymes, and myocardial markers in elderly patients undergoing hysterectomy.

**Methods:**

Group A and group B were established randomly regarding a total of 126 elderly patients who underwent an elective hysterectomy. Patients in group A were given full anesthesia with sevoflurane, and patients in group B were given anesthesia with intravenous anesthesia. The operation time, anesthesia time, and recovery time in Postanesthesia Care Unit (PACU) were compared; plasma cortisol concentration, hemodynamics, serum myocardial enzymes, and myocardial markers were detected and compared between the two groups of patients before anesthesia (*T*_0_), after anesthesia (*T*_1_), and after surgery (*T*_2_).

**Results:**

Group A observed a longer extubation time and recovery time in PACU than group B (*P* < 0.05). Results show a lower systolic blood pressure (SBP), diastolic blood pressure (DBP), heart rate (HR), and plasma cortisol concentration of *T*_1_ by comparison with those of *T*_0_ (*P* < 0.05), but no significant difference remains in terms of intergroup SBP, DBP, and HR (*P* > 0.05), and there was no interaction effect of groups and time (*P* > 0.05). The two groups showed no great disparity in the levels of lactate dehydrogenase (LDH), aspartate transaminase (AST), creatine kinase (CK), and CK-MB as a subtype of CK before surgery between the two groups of patients (*P* > 0.05). After surgery, LDH, AST, CK, and CK-MB levels in both groups were witnessed a surge, in which group A obtained higher levels of LDH, AST, CK, and CK-MB (all *P* < 0.05).

**Conclusion:**

Total intravenous anesthesia will not increase the hemodynamic fluctuation of elderly patients undergoing hysterectomy and can reduce the damage to the myocardium of patients with surgical trauma, which can protect the myocardium of elderly patients to a certain extent, so it can be adopted as the optimal anesthesia protocol for surgery.

## 1. Introduction

Hysterectomy, a frequently adopted gynecologic surgery, is the principal treatment for treating female reproductive tumors, adnexal lesions, and uterine prolapse. However, being accompanied by merging multiple-organ disorders or organ metabolic disorders, most elderly patients are highly sensitive to anesthetics with poor stress tolerance, which leads to apparent hemodynamic changes during surgery and a propensity for perioperative myocardial ischemia events. Moreover, the therapeutic effect and postoperative recovery can be ensured only by using proper anesthetic methods [1–3]. Therefore, it is of great significance to find a safe and effective anesthetic method that is more suitable for elderly patients to improve perioperative hemodynamics and myocardial function. Full inhalation anesthesia (FIA) and total intravenous anesthesia (TIVA) are two safe and full-fledged anesthesia programs commonly adopted in surgery. Previous studies have exhibited the advantages and disadvantages of the two anesthesia programs [4, 5], but there are no reports of the two anesthesia programs during hysterectomy in elderly patients. Therefore, this paper is to probe into the effects of the two anesthetic modalities on hemodynamics and myocardial function in elderly patients undergoing hysterectomy by monitoring changes in systolic blood pressure (SBP), diastolic blood pressure (DBP), heart rate (HR), serum myocardial enzymes, and myocardial markers, to provide an effective reference for the clinical selection of anesthetic modalities in elderly patients undergoing hysterectomy. It is reported below.

## 2. Information and Methods

### 2.1. General Information

A total of 126 female patients who underwent an elective hysterectomy in our hospital from June 2017 to June 2019 were enrolled as subjects. Inclusion criteria are as follows: (1) patients received hysterectomy in our hospital due to reproductive system tumor, uterine adnexal disease, uterine prolapse, and other diseases; (2) age ≥ 60; (3) American Society of Anesthesiologists (ASA) I~II patients; (4) patients were fully informed and voluntarily participated in this study; and (5) patients not involved in other studies during this study. Exclusion criteria are as follows: (1) severe anemia; (2) with cognitive impairment; (3) with abnormal mental state; (4) myocardial ischemic events in the last six months; and (5) allergic to the anesthetic.

Group A and group B were established with the random number table method regarding the subjects enrolled. The two groups presented no significant difference in general data (*P* > 0.05), which was comparable. Approval of this study was obtained from the Medical Ethics Committee of our hospital, and all subjects provided informed consent. The study strictly complied with the requirements of the ethics committee, with the ethics committee number of 2016-11-15.

### 2.2. Methods

Firstly, venous passage was opened after the two groups of patients entering the operating room. Electrocardiogram (ECG) monitor was connected to timely monitor the patient's SBP, DBP, and HR; the bispectral electroencephalogram index (BIS) monitor was connected to monitor the depth of anesthesia. After five minutes of oxygen intake, a tracheal tube was inserted for mechanical ventilation. Patients of group A were induced with anesthetic using 2 mg/kg propofol (manufacturer: Fresenius Kabi Deutschland GmbH; specification: 20 mL: 0.2 g; Chinese medicine standard: J20110055), 0.9 mg/kg rocuronium bromide (manufacturer: Zhejiang Xianwei Pharmaceutical Co., Ltd.; specification: 1 mL: 5 mg; Chinese medicine standard: H20093186), 0.5 *μ*g/kg sufentanil (manufacturer: Yichang Renfu Pharmaceutical Co., Ltd.; specification: 1 mL: 50 *μ*g; Chinese medicine standard: 1150312), inhaled sevoflurane (Manufacturer: Lunan Beit Pharmaceutical Co., Ltd.; Specification: 100 mL; Chinese medicine standard: H20080681) for maintenance, and the end-expiratory concentration was maintained at 0.7~0.3 minimum alveolar concentration (MAC). Patients of group B were induced with anesthesia using 2 mg/kg propofol, 0.9 mg/kg rocuronium bromide, and 0.5 *μ*g/kg sufentanil, and plasma target-controlled injection was carried out with 3~5 *μ*g/mL propofol combined with 3~5 ng/mL sufentanil, with an intermittent injection of rocuronium maintained. Both groups stopped the administration of rocuronium and sufentanil 0.5 h before the completion of the operation and immediately stopped when the pneumoperitoneum was closed. Group A was eluted with oxygen flow > 6 L/min after stopping the administration. Both groups maintained mechanical ventilation, and extubation was carried out after the emergence of extubation indication.

### 2.3. Observation Indicators

(1) After collecting the venous blood samples, the hemodynamic indexes such as SBP, DBP, and HR, and plasma cortisol concentration before anesthesia (*T*_0_), 1 h after anesthesia (*T*_1_), 1 h after operation (*T*_2_) of the two groups of patients were recorded. The plasma cortisol concentration was detected by applied radioimmunoassay. (2) Approximately 5 mL of venous blood was extracted before and after the operation, respectively. Serum myocardial enzymes (LDH, AST, CK, and CK-MB as a subtype of CK) were detected by an Italian BT-224 semiautomatic analyzer

### 2.4. Statistical Processing

SPSS 22.0 was adopted for statistical analysis. The quantitative data were described by frequency or percentage. *χ*^2^ inspection was carried out. Quantitative data consistent with the normal distribution were represented by (*x* ± SD). A *t*-test was adopted for a two-group comparison. Repeated measurement analysis of variance was applied to compare the data at different time points between groups. *P* < 0.05 stands for striking difference.

## 3. Results

### 3.1. Comparison of General Information, Operation, and Anesthesia Time

The general information such as age and ASA grading, operation, and anesthesia time between the two groups were compared shown in Table 1.

### 3.2. Comparison of Hemodynamic Indexes between the Two Groups

**Figure 1:**
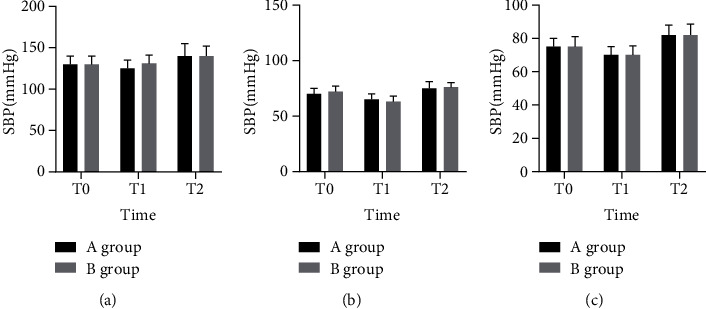
Changes of hemodynamic indexes at different time points of the two groups. (a) SBP of patients in the two groups at different time points. (b) DBP of patients in the two groups at different time points. (c) HR of patients in the two groups at different time points.

There was no statistical difference of SBP, DBP, and HR between the two groups (intergroup effects: *F*_SBP_ = 0.021, *F*_SBP_ = 0.886; *P*_SBP_ = 0.937, *P*_DBP_ = 0.334; *F*_HR_ = 0.107, *P*_HR_ = 0.744; *F*_Plasma cortisol_ = 0.217, *P*_Plasma cortisol_ = 0.773); SBP, DBP, and HR of patients in both groups had the trend of changing depending on time (time effects: *F*_SBP_ = 38.891, *P*_SBP_ = 0.796; *F*_DBP_ = 0.578, *P*_DBP_ = 0.562; *F*_HR_ = 0.138, *P*_HR_ = 0.872; *F*_Plasma cortisol_ = 0.337, *P*_Plasma cortisol_ = 0.813) shown in Table 2 and Figure 1.

### 3.3. Comparison of the Levels of Serum Myocardial Enzymes and Myocardial Markers between the Two Groups

The comparison of LDH, AST, CK, and CK-MB levels before operation between the two groups was not statistically significant (*P* > 0.05). After surgery, LDH, AST, CK, and CK-MB levels in both groups were witnessed a surge, in which group A obtained higher levels of LDH, AST, CK, and CK-MB (all *P* < 0.05) (Tables 3 and 4).

## 4. Discussion

Hysterectomy is a common gynecologic procedure for treating uterine diseases, with general anesthesia as the most frequently adopted anesthetic method. The anesthesia, as well as the tracheal intubation after anesthesia, surgery, and other operations will result in certain trauma, which triggers oxidative stress responses in the patient's body and blood vessels. Vascular stress response will change the patient's hemodynamics, resulting in elevated blood pressure and increased heart rate during and after surgery, which not only increases the risk of surgery but also hinders the postoperative recovery of patients [6, 7]. Due to the decline of body immune function in elderly patients, the tolerance to trauma is decreased, the hemodynamic changes are easier during anesthesia and surgery, and the risk of postoperative adverse reactions is also greater [8]. Besides, surgical trauma can also lead to myocardial ischemia. Li et al. [9] also revealed a higher risk of visceral injury in surgical centers in elderly patients. Therefore, it is of great significance to find an anesthetic method more suitable for elderly patients undergoing hysterectomy to stabilize their perioperative hemodynamics and reduce the occurrence of adverse cardiac events.

FIA and TIVA are the two commonly adopted surgical anesthesia methods at present. FIA is the use of volatile gas or liquid drugs, which is through the patient's respiratory tract inhalation of the body to exert its anesthetic effect [9]. Sevoflurane, one of the most frequently adopted drugs in the whole process of inhalation anesthesia, can inhibit the circulatory system of the body and has the effect of lowering hypertension during operation and reducing cardiac output [10]. Intravenous anesthesia refers to the maintenance of anesthesia through either continuously or intermittently intravenous infusion of various short-acting intravenous anesthetics after intravenous induction [11]. Propofol and sufentanil are the two drugs that are often adopted in total intravenous anesthesia. Propofol is a new, rapid, and short-acting intravenous anesthetic that can not only induce anesthesia in a short period but also avoid hindrance on the recovery of various functions after surgery, without increasing the incidence of postoperative adverse reactions [12]. Sufentanil is a *μ* receptor agonist, which has a strong analgesic effect and high liposolubility and can quickly succeed in anesthesia after entering the body, without obvious accumulation in the patient's body, which will not prolong the postoperative recovery time [13]. The comparison of the effect of sevoflurane inhalation anesthesia with propofol combined with sufentanil on hysterectomy in elderly patients revealed that there were lower SBP, DBP, and HR of *T*_1_ by comparison with those of *T*_0_ (*P* < 0.05), which suggests that both methods can stabilize hemodynamics after anesthesia. The results of repeated ANOVA presented that no significant difference remains in terms of intergroup SBP, DBP, and HR (*P* < 0.05), and there was no interaction effect of groups and time (*P* > 0.05), implying that the inhibitory effect of the two anesthetic methods on the circulatory system in elderly patients was similar.

Surgical trauma and anesthesia can directly affect the myocardial enzymes. There was no significant difference in LDH, AST, CK, and CK-MB levels before surgery between the two groups of patients (*P* > 0.05). After surgery, LDH, AST, CK, and CK-MB levels in both groups were higher than those before surgery, implying that surgical trauma does cause some damage to the heart muscle. However, LDH, AST, CK, and CK-MB levels of patients who underwent propofol combined with sufentanil under intravenous anesthesia of group B were lower than those in group A (all *P* < 0.05), implying that propofol combined with sufentanil can mitigate myocardial damage in elderly patients by intravenous anesthesia. The reason probably is that as an intravenous anesthetic, propofol has strong antioxidant properties and can directly act on oxygen free radical calcium channels and neutrophils, which can prominently inhibit calcium channels and reduce neutrophil activity, so it can abate the stimulation and injury of patients with anesthesia, thus reducing the perioperative myocardial damage in elderly patients [14, 15]. However, this study is limited by the sample size and experimental conditions, and the study of drug proportion and experimental results still need more experiments for verification.

To sum up, TIVA will not increase the fluctuation of hemodynamics in elderly patients after hysterectomy and can reduce the damage of surgical trauma to the myocardium of patients to a certain extent, to protect the myocardium of elderly patients, which can be adopted as an optimal anesthetic scheme for elderly patients undergoing hysterectomy.

## Figures and Tables

**Table 1:** General information, operation, and anesthesia time of the two groups.

	*n*	Age (years)	ASA grading		Operation time (min)	Extubation time (min)	Recovery time in PACU (min)
			I	II			
Group A	63	60-75 (67.71 ± 4.12)	29 (46.03%)	34 (53.97%)	95.2 ± 18.9	15.2 ± 5.3	34.5 ± 10.8
Group B	63	61-76 (67.89 ± 4.30)	30 (47.62%)	33 (52.38%)	94.9 ± 17.9	13.8 ± 4.9	24.6 ± 8.3
*t*/*χ*^2^		0.239	0.032		0.365	3.647	5.698
*P*		0.810	0.858		0.369	0.001	0.002

**Table 2:** Changes of hemodynamic indexes at different time points of the two groups ($\bar{x}\pm s$).

	Groups	Cases	*T*_0_	*T*_1_	*T*_2_
SBP (mmHg)	Group A	63	127.56 ± 13.12	118.65 ± 14.26	135.96 ± 15.83
	Group B	63	127.26 ± 13.07	119.71 ± 14.69	134.56 ± 15.62
DBP (mmHg)	Group A	63	81.59 ± 8.96	76.52 ± 7.82	84.19 ± 8.62
	Group B	63	83.62 ± 8.92	76.25 ± 7.63	84.97 ± 8.96
HR (each time/min)	Group A	63	76.31 ± 7.58	70.85 ± 6.67	83.47 ± 7.85
	Group B	63	76.94 ± 8.19	71.34 ± 7.58	83.14 ± 9.01
Plasma cortisol	Group A	63	291 ± 35.3	304.9 ± 40.9	257.6 ± 61.4
	Group B	63	290.1 ± 35.1	300.7 ± 37.9	256.4 ± 54.3

**Table 3:** Comparison of LDH and AST levels before and after surgery in the two groups ($\bar{x}\pm \text{s}$).

Group	LDH (U/L)		*t*	*P*	AST (U/L)		*t*	P
	Before the operation	After the operation			Before the operation	After the operation		
Group A	231.95 ± 18.64	246.18 ± 19.57	4.179	<0.001	32.59 ± 7.48	46.25 ± 8.01	9.893	<0.001
Group B	229.68 ± 18.59	238.28 ± 19.18	2.556	0.012	32.05 ± 7.51	41.85 ± 7.92	7.127	<0.001
*t*	0.684	2.288			0.404	3.100		
*P*	0.495	0.024			0.687	0.002		

**Table 4:** Comparison of CK and CK-MB levels between two groups of patients before and after the operation ($\bar{x}\pm s$).

Group	CK (U/L)		*t*	*P*	CK-MB (U/L)		*t*	*P*
	Before the operation	After the operation			Before the operation	After the operation		
Group A	119.16 ± 12.63	168.65 ± 15.62	19.561	<0.001	16.15 ± 5.68	21.95 ± 6.24	5.456	<0.001
Group B	117.33 ± 12.95	159.33 ± 14.96	16.852	<0.001	16.92 ± 5.16	19.57 ± 5.06	2.910	0.004
*t*	0.803	3.420			0.796	2.351		
*P*	0.424	<0.008			0.427	0.020		

## Data Availability

All datasets are available from the corresponding author upon reasonable request.
